# Addressing ethical issues related to prenatal diagnostic procedures

**DOI:** 10.1186/s40748-023-00146-4

**Published:** 2023-02-03

**Authors:** Dan Kabonge Kaye

**Affiliations:** 1 Department of Obstetrics and Gynecology, Makerere University College of Health Sciences, Kampala, Uganda; 2 Forgarty African Bioethics Postdoctoral Fellow at Johns Hopkins Berman Institute of Bioethics and the School of Public Health, Baltimore, MD USA

**Keywords:** Prenatal diagnostic procedures, Preconception counseling, Ethical issues

## Abstract

**Background:**

For women of advanced maternal age or couples with high risk of genetic mutations, the ability to screen for embryos free of certain genetic mutations is reassuring, as it provides opportunity to address age-related decline in fertility through preimplantation genetic testing. This procedure has potential to facilitate better embryo selection, improve implantation rates with single embryo transfer and reduce miscarriage rates, among others, yet confers some risk to the embryo and additional costs of assisted reproductive technology. This raises questions whether, when and which patients should receive routine PGT-A prior to embryo transfer.

**Discussion:**

Prenatal diagnostic procedures refer to tests done when one or both genetic parents has a known genetic disorder (or has worries about the disorder) and testing is performed on them, their gametes or on the embryos to determine if the latter is likely to carry a genetic disorder. PGT is used to identify genetic defects in gametes or embryos (often created through in vitro fertilization (IVF). The procedures generate immense potential to improve health and wellbeing by preventing conception or birth of babies with undesirable traits, life-limiting conditions and even lethal conditions. However, they generate a lot of information, which often may challenge decision-making ability of healthcare providers and parents, and raise ethical challenges.

**Conclusion:**

Prenatal diagnostic procedures have potential to address uncertainty and risk of having a child affected with a genetic disease. They, however, often raise own uncertainty and controversies, whose origin, manifestation and related ethical issues are presented. There is need to develop individual and couple decision support tools that incorporate patients’ values and concerns in the decision-making process in order to promote more informed decisions, during counseling.

## Background

Prenatal diagnostic testing include tests done on individuals, their gametes, embryos or unborn fetus with the purpose of detecting disorders, including certain hereditary or spontaneous genetic disorders. Prenatal genetic testing (PGT) is indicated in couples carrying balanced chromosomal translocation, since about half of the embryos would have chromosomal abnormalities, ad thereby contribute to implantation failure, early miscarriage or fetal anomalies. Such procedures include routine ultrasonography and certain blood tests (as part of routine prenatal care procedures) as well as, or as a precursor, to more invasive prenatal genetic tests (such as chorionic villus sampling, amniocentesis, and percutaneous umbilical blood sampling) [1]. The procedures may include genetic analysis of artificially fertilized embryos to select an embryo with a desired genotype before it is implanted. In in-vitro fertilization (IVF), PGT procedures are used to screen in-vitro fertilized embryos for their potential success in uterine implantation, in an attempt to improve pregnancy rates, and are indicated in cases of male infertility, advanced maternal age and recurrent miscarriage [2]. The goal of such testing is to determine better embryo selection, improve implantation rates with single embryo transfer and reduce miscarriage rates, thereby addressing age-related decline in fertility [1–3]. In addition, PGT reduces the risk of conceiving a child with genetic disorders, thus has potential to reduce rates of elective pregnancy termination for fetal/embryo abnormalities as the indication [3, 4]. A strategy to combine screening for aneuploidy embryos with the routine in vitro fertilization (IVF) procedure is called preimplantation genetic testing of aneuploidy (PGD-A). This paper addresses the questions of whether, when and which patients should receive PGT-A prior to embryo transfer, and the implications for informed decision making. The more invasive tests are conducted when couples have an increased risk of a chromosomal disorder (particularly when the woman is 35 or older), or having a baby with the congenital anomaly such as a neural tube defect. [1]. The more invasive genetic tests and procedures conducted on the gametes and early embryos are referred to as pre-implantation genetic testing (PGT) [2, 3]. These tests are routinely provided for couples who seek assisted reproductive technologies in Uganda.

Many pregnant women and couples are offered prenatal diagnostic procedures on request or are advised to have them conducted routinely. Before accepting or requesting for the tests, couples should discuss the risks with their healthcare practitioner and weigh the potential risks against their need to know, and should consider the effects of knowing the results on their wellbeing, as well as the implications of the knowledge gained on healthcare decisions [1] For some couples, the risks of undergoing the tests or knowing the results outweigh the benefits of knowing whether their baby has a genetic or chromosomal disorder, and may choose not to be tested [1, 3]. There is limited information on individual and couple decision-making processes for prenatal diagnostic procedures including PGT [1, 3]. Yet the increasing technical complexity and evolving options for PGT have implications for information processing and decision making for couples faced by decision regarding whether to authorize the tests and what decisions to take after knowing the results [4–6].

The factors which couples consider in decision-making could include motivation by prospects of a healthy, genetic-variant-free child, ability to commit time, financial resources and emotions, considerations for what would be done to the unused embryos or whether it is right to discard them, and the patients’ trust in and acceptance of results of the available technologies [5]. Such decisions are always complex for individuals and couples [5]. Not only is there scanty data on PGT decision-making processes, the available data is inconsistent, partly from failure to use validated instruments [5]. Couples' decision-making involves three dynamic dimensions: cognitive appraisals (subjective interpretation made by an individual to stimuli in the environment), emotional responses (the emotions an individual goes through after receiving information), and moral judgments (the process by which individuals define what is right or wrong) [6]. All these factors further compound the uncertainty for couples beset with making decisions about PGT. This paper analyses the issues of uncertainty that characterize prenatal diagnostic procedures in general and PGT in particular, with suggestions on how these could be mitigated, prevented or addressed.

## Main Text

### Preimplantation genetic testing

Preimplantation genetic screening (PGS) and preimplantation genetic diagnosis (PGD) are terms traditionally used to describe genetic testing of embryos before a pregnancy is established [2, 3]. While PGS was used to refers to screening for chromosomal disorders (such as Down syndrome), PGD was used to screen for genetic defects involving a single gene (such as cystic fibrosis) [3]. Currently, the term Preimplantation genetic testing (PGT) encompasses preimplantation genetic screening (PGS) and preimplantation genetic diagnosis (PGD), and is used to refer to all the series of tests performed to analyze the DNA from oocytes (polar bodies) or embryos (cleavage stage or blastocyst) for HLA-typing or for determining genetic or chromosomal disorders [2]. PGT includes PGT for aneuploidies (PGT-A) (previously called PGS or preimplantation genetic screening); PGT for monogenic/single gene defects, including autosomal recessive, autosomal dominant, and X-linked conditions (PGT-M); and PGT for chromosomal structural rearrangements (PGT-SR) (previously called PGS translocation) [2]. PGT is extremely useful in several situations: First, PGT is used in screening for couples in which one or both partners are carriers of an inherited genetic disorder, or suspect to have high risk of such inheritable disorders [2–4]. Secondly, PGT improves success rates of in vitro fertilization by ensuring the transfer of euploid embryos that have a higher chance of implantation and resulting in a live birth [3, 4]. Here, PGT enables the identification of embryos with specific disease-causing mutations and therefore transfer of unaffected embryos. For instance, PGT may be used where a couple carries a gene for specific disorders (such as hemoglobinopathies), where genetic testing is conducted on the embryo before implantation. Thirdly, PGT may be used to identify chromosomally normal embryos to transfer so as to achieve a normal pregnancy, after considering other factors such as high maternal age, the number and quality of embryos, the results of the embryo biopsies, and other fertility-related factors [3–5]. The technique provides a practical alternative to preconception diagnosis so as to prevent termination of pregnancy in couples with a high risk for offspring affected by a sex-linked genetic disease, mono-genetic disorders or autosomal dominant diseases such as myotonic dystrophy, Huntington’s disease and Marfan’s syndrome. For sex-linked diseases, the embryos are tested to ascertain the sex so that only female embryos are transferred. Genetic analysis may also be conducted at the single-cell level, where first and second polar bodies from oocytes or blastomeres from cleavage-stage embryos are assessed [3–5].

There are three main groups of disorders for which PGT is indicated. X-linked diseases are inherited from a mother who is a carrier, and are caused by an abnormal X chromosome and manifest in sons, who do not inherit the normal X chromosome from the father [7]. Since, the X chromosome is transmitted to offspring/embryos through the mother, affected fathers have sons who are not affected, while their daughters have a 50% risk of being carriers if the mother is asymptomatic [7]. Sex-linked recessive disorders include hemophilia, fragile X syndrome, most neuromuscular dystrophies [7]. PGT may be used to identify single gene defects such as cystic fibrosis, Sickle disease, sickle cell anemia, and Huntington disease, where the abnormal gene may be detectable with molecular techniques using polymerase chain reaction (PCR) amplification of DNA from a single cell [7]. Indications for prenatal diagnosis include women of advanced maternal age, history of an affected family member, couples with history of recurrent pregnancy loss, male partner with severe male factor infertility, and couples with repeated IVF failure [7]. PGT can decrease the risk of IVF failure by selecting chromosomally normal embryos with a higher chance of implantation and eventual pregnancy [3, 4, 7].

### Prenatal diagnostic procedures during pregnancy

There are several diagnostic procedures that can be conducted during the first and second trimester of pregnancy. Screening during the first trimester usually consists of: 1) blood tests to measure levels of pregnancy-associated placental protein A (produced by the placenta) and beta-human chorionic gonadotropin in the pregnant woman’s blood; 2) Ultrasonography to assess for fetal nuchal translucency). Both tests are used to screen for Down Syndrome and certain other chromosomal disorders.3) Cell-free fetal nucleic acid [cfDNA] testing) may be done to determine the risk of Down syndrome and some other chromosomal disorders in couples with a high risk of having a fetus with a chromosomal disorder. One advantage of first-trimester screening is that with earlier results, abortion, if desired, can be done earlier, when it is safer.

During the second trimester, markers in the pregnant woman’s blood can be measured (and together with ultrasonography, can evaluate the risk that the fetus will have certain disorders. The tests include measurement of Estriol: formed from precursor substances produced by the fetus, Human chorionic gonadotropin (produced by the placenta), Inhibin A (produced by the placenta) and alpha-fetoprotein (a high level of which may indicate an increased risk of having neural tube defects of the brain or spinal cord (spina bifida), defects of the abdominal wall and intrauterine fetal growth restriction.

### Uncertainty related to prenatal diagnostic procedures

Prenatal diagnostic procedures in general and PGT in particular generate a lot of uncertainty regarding decision-making. Individuals and couples think critically about uncertain information, contend with conflicting emotions, and combine moral perspectives into their decision-making about whether or not to accept PGT [1, 6]. Decisional factors related to values about conception, disability, pregnancy termination, past pregnancy experiences, optimism toward technology and cost play a critical role in the decision-making process for PGT [8]. Other factors important in decision-making include opportunities for expanded carrier screening prior to in-vitro fertilization (IVF) itself, maternal age and knowledge about IVF and PGT [8]. There is limited opportunity to access decision support tools that incorporate patients’ values and past experiences in the decision-making process to promote a more informed decision [8]. Cost implications for PGT are critical: for instance, the cost to attain a 50% likelihood of a normal blastocyst may be up to 10 times higher older women (aged above 40 years) with low Anti-Mullerian hormone (AMH) levels when compared with the young women with high AMH values [9]. As a state, uncertainty may vary from falling short of certainty to an almost complete lack of conviction or knowledge specifically about an outcome or result, with inability to make a decision, or unwillingness to believe without conclusive evidence [10]. Uncertainty in healthcare is experienced by patients and healthcare professionals in differing ways, motivates diverse actions, and elicits diverse responses [11–13]. All screening tests harbor the prospect that uncertain information could arise. The data generated from prenatal diagnostic procedures implies that data of uncertain diagnostic significance, uncertain prognosis, or meaning that changes over time (as more knowledge becomes available) may be generated [6, 8–10], with diverse ethical, legal and clinical implications. Uncertainty in prenatal diagnostic procedures is particularly related to communication (or failure to communicate) uncertain information [14–17].

Often times, families get concerned about conceiving and delivering a child who may have preventable genetic disorders or disabilities [17]. The parents may consider options such as pre-conceptional counseling, genetic or genomic testing and screening and prenatal non-invasive or invasive diagnostic procedures. The goal of all these is to prevent or identify fetuses that are affected with a certain undesirable trait so that parents may decide whether to conceive, terminate the pregnancy, or (if they opt to continue the pregnancy) to have the pregnancy as a high-risk pregnancy. The latter option involves preparations for pregnancy and eventual childbirth after appropriate counseling and support) [7, 17, 18]. Where the pregnancies are unaffected, the parents are given reassurance, which is beneficial for pregnancies with a high a priori risk [17, 18].

There are several ethical issues related to use of this technology, often related to ethical implications of the results generated by the technology, particularly the potential for harm that may arise from decision-making. The technological advances may enable couples prevent birth of a child with undesirable defects, and from the societal value, can reduce the burden of genetic and hereditary disorders [17, 18]. For instance, genetic testing may identify hereditary disorders such as hemoglobinopathies. Genetic testing can also be used to determine severity of disease [17]. For instance, there are over 2,000 different mutations in the gene that causes cystic fibrosis [7]. Not all of them cause disease, and of those that are disease-causing, different mutations cause different levels of severity of disease [7]. Genetic testing can be performed as part of a couple’s preconception care, usually carrier screening because one or both parents have a family history or an increased risk for having a particular mutation.

Prenatal diagnostic procedures generate a lot of data, which is the source of uncertainty.

Often, the genetic screening creates more uncertainty and raises ethical issues related to how to handle the information generated. Screening tests primarily target identification of chromosome disorders, notably Down syndrome (through a combination of maternal age, maternal serum biochemical tests, and fetal ultrasound) and single gene disorders with Mendelian patterns of inheritance, identifiable by screening for carrier status (such as hemoglobinopathies) [7, 17, 18]. However, few pregnancies can be identified as high-risk, depending on the population screened and the test protocols, yet the latter can be very costly [7]. For X-linked disorders, half of the discarded male embryos are normal, while half of the female embryos transferred may be carriers of the condition [19]. For Duchenne muscular dystrophy and fragile X syndrome, even half of female embryos may be affected [19].

In non-disclosure PGT as used for Huntington’s disease and some late-onset diseases, patients may not wish to know their carrier status but want to have disease-free offspring [19], such that embryos are tested without revealing any of the details of the diagnosis [19]. Using Human Leukcocyte antigen (HLA) testing, PGT may be undertaken to select embryos not affected with a disease, such as Fanconi anemia, but which have the same HLA type as an affected sibling [19]. In this case, a child is conceived to be used as a treatment for a sibling, thereby breaching the Kantian imperative that a person should never be used as a means. There is concern that that children conceived for the benefit of their siblings are not valued in their own right [19, 20]. Should the latter child be informed that they were conceived primarily to provide therapy for an elder sibling? The counter argument is that all children may be valued, that the HLA stem cell child donor may even be more valued for having contributed to the health of a sibling, and that it may not harm either sibling if they are informed at the appropriate age [19, 20]. Also, where the screening test is inconclusive, a definitive diagnosis through invasive tests, such as amniocentesis or chorionic villus samples (CVS) and karyotyping may be necessary [7, 19, 20], with additional cost and potential risk of miscarriage or maternal complications.

Additional non-invasive tests include screening for fetal cell-free DNA and RNA in maternal serum [20, 21], where chromosomal aneuploidies such as Down’s syndrome can be identified by an abnormal ratio of different chromosomes [20]. These tests can be used to screen for several disorders [20–22]: a) presence or absence of male-specific (Y-chromosome) sequences may be used to assess fetal sex, useful for rare sex-linked inherited diseases that affect only one sex; b) Presence or absence of the RhD gene may be used to assed fetal RhD blood group status, which is useful for RhD-negative mothers at risk of RhD incompatibility reactions caused by RhD positive fetuses; c) presence (or absence) of corresponding sequences especially those inherited from the father of the fetus, may identify inherited genetic diseases. Prenatal screening and diagnosis may raise uncertainty. In these contexts, the options are to not get pregnant, continue a pregnancy while knowing the potential risks, terminate a pregnancy after an informed decision, or (in the case of a preimplantation genetic diagnosis), determining whether and which embryos to implant embryos or discard.

### Types and implications of uncertainty posed by prenatal diagnostic procedures

Despite these potential benefits, technological advances have potential for causing more uncertainty and even harms. There is the ethical issue of the technological imperative [23], which suggests that since the technology is available, there are compelling reasons to use it, yet this needs to balanced again the patients’ best interests. Before conducting the tests, the doctors and their patients need to think through what the results might mean, and what decisions may be considered once the results become available, while considering the best interest of the unborn child as well. This standard may be difficult to apply and may not provide meaningful practical guidance in certain situations. To compound the uncertainty, it may be difficult to precisely define the ‘‘best interest’’ of an unborn child, how to determine best interests may be controversial, the nature of interests may be complex, and it is unclear what weight the best interest should have in the decision-making compared to social values [24].

#### Requirements for informed consent

There are additional challenges posed by this uncertainty. First, requirements for an informed consent imply that couples should have sufficient relevant information about the procedures, and that they understand potential risks and benefits, the decisions are voluntary and information on available options is discussed [24]. Where there are few genetic counselors with current information on screening protocols, it is unclear whether pregnant women are able to make informed choices about prenatal screening [7, 20, 24]. Where prenatal screening is part of routine prenatal care, couples may be unlikely to be offered opportunity for deliberate decisions about having prenatal screening [24]. Secondly, there is uncertainty related to what to do with the embryos that have disorders, leading to an ethical challenge of embryo wastage.

#### Accuracy and reliability of prenatal diagnostic procedures

There is uncertainty (and ethical issues) related to the accuracy and reliability of the tests: how good the test has to be to be used in different contexts, considering false positives, false negatives and the prevalence of the disorder under screening [7, 20]. How much risk is the couple willing to take that the test is wrong and that they will conceive (or have) a child that might carry the genetic traits? Also, there is the uncertainty of penetrance for genetic diseases [7, 20, 24]. How certain must we be that the mutation will cause disease? For instance, different mutations of the cystic fibrosis gene have different risks of causing the disease, and some mutations for other diseases may cause disease at certain times (or situations) and not in others, making it difficult to predict whether a particular mutation will cause disease. This uncertainty becomes particularly important where one considers to perform an invasive test such as chorionic villus sampling and amniocentesis, which carry significant risks to the fetus or the pregnancy.

#### Uncertainty regarding sex selection and paternity testing

In addition, early sex identification available through the non-invasive prenatal diagnostics, may encourage sex selection by couples who would not have resorted to ultrasound or invasive tests for this purpose [19]. Use of PGT for sex selection unrelated to disease is controversial, as it leads to failure to implant normal embryos when they are found to be of the undesired sex [20]. This raises moral objections due to danger of sex discrimination. This is particularly problematic in societies which have a strong preference for boys [7, 20]. Besides, the increased identification of fetuses with disorders, even borderline disorders, has the potential to increase numbers of pregnancy termination for medical reasons [18, 20, with potential increase in demand (from opponents of abortion) for restrictions on the women’s right to terminate pregnancy [7, 20]. Another area of uncertainty and controversy that raises ethical concerns relates to potential use of non-invasive prenatal diagnostic for paternity testing [25, 26]. This is ethically problematic in cases where paternity is uncertain and a woman uses results of such tests to opt for pregnancy termination [26].

#### Uncertainty of related to consideration of giving birth to individuals with disability

The ethical issues related to completeness, accuracy, and bias in the information communicated to couples is particularly important for a prenatal diagnosis of Down syndrome [27]. The real choice about giving birth to a child with a genetic or chromosomal disorder depends on more than the perceived availability of care and support for a child with a disability [27]. Some physicians and counselors often focus on the negative aspects of the associated disability, rather than providing all information for couples to make an informed decision [25, 28]. Some parents may not mind having a child with the disability as long as complete information is availed to them to make informed decisions [25, 28]. Increased testing accompanied by pregnancy terminations could potentially reduce the incidence and prevalence of some genetic or chromosomal disorders associated with disability [20, 27]. Yet decline may negatively change public attitudes towards the hereditary disabilities specifically and all disability or handicapped in general, thereby reducing the moral worth of individuals with disability [25, 28], especially where prenatal diagnostics are performed primarily to prevent birth of “disabled” babies [25]. This may lead to reduced understanding or support for affected individuals and their families [25, 28].

Prenatal genetic testing at beginning of life raises controversy in certain situations. Questions arise on how bad (lethal, severe, or disabling) a condition should be in order to warrant testing. Disability communities differ regarding the ways in which they think about this question. Performing genetic testing to try to avoid a certain condition implies that the life of a person with that condition is not worth living [7]. How people think about that question depends partly on the type of condition being discussed, at what stage in the continuum from preconception to preimplantation to prenatal period the testing is done, and what test is performed. There is a big debate in the ethics community, society and popular media about the appropriateness of prenatal screening for disorders such as Down syndrome during pregnancy, and whether it's proper to take any actions to terminate pregnancies based on screening results [7, 25, 28]. There is also an ethically problematic issue of equity, related to access [20], as procedures may be costly to some population, especially where routine screening is not available, consequently adding to the already existing inequalities in access to care. This relates mainly to expanding testing and control over non-essential characteristics (those not required for life) in offspring. However, different individuals and communities have diverse personal, religious, ethical, and moral norms views and values, which should be respected must be given by healthcare professionals when discussing the performance of PGT for sex selection.

#### The uncertainty posed by mosaicism

Mosaicism describes presence of more than one type of cell in an embryo. For instance, an embryo may have some of the cells with 46 chromosomes, while other cells have 47 chromosomes, as in mosaic Down syndrome. In this condition, about 95% of affected individuals have trisomy 21 (with an extra chromosome in every cell), while 3–4% have translocation Down syndrome (where all or part of the extra chromosome-21 is attached to another chromosome), and 1–2% are mosaic (where some cells have 46 chromosomes and others 47 chromosomes). Mosaicism is usually described as a percentage; however, the percentage of mosaic cells may differ in the different tissues, implying that the percentage of mosaicism detected may depend on the tissue assessed [29]. Besides, the degree of mosaicism may vary with the stage of development at which embryo biopsy is conducted [30], as self-correction may occur as the embryo develops.

#### Uncertainty related to timing of prenatal genetic testing

Another area of uncertainty is when to test. Where a test can be performed at different stages of the continuum (preconception, pre-implementation, during pregnancy or postnatal), there is uncertainty about choosing the most appropriate time or tests. One may need to consider how bad (in terms of disability or life limiting) the condition be in order to warrant testing. How good should the test be? One wonders whether the stage (on the continuum) should matter. One relevant question is what types of conditions it is appropriate to test for [25, 28]. One may consider whether the condition is lethal, serious life-limiting or just mildly disabling. One may also consider whether the medical conditions may or may not develop later in pregnancy or later in life, or whether if develops, it is life limiting, or may even never develop.

#### Additional uncertainty related to mandatory newborn screening

A key ethical and legal issue relates to the mandatory nature of newborn screening in some countries. It is relatively easy to justify mandatory newborn screening for conditions such as Phenylketonuria (PKU), because if the condition was identified before the baby becomes symptomatic, the baby would be treated to achieve a good outcome. The challenge is in screening for disorders where there is not enough evidence on effectiveness of the screening, and if this is mandatory, whether some form of parental consent is necessary. And one solution to address the above challenge is tiered screening, where there is mandatory screening for the conditions such as PKU (where there is good evidence to support population-wide screening and there's good treatment available for infants that are identified pre-symptomatically) and selected screening for other conditions where evidence is not as good, or where the potential benefit of identifying these babies in infancy is less clear. For the latter, parental consent may be necessary.

How to provide adequate counseling for mandatory screening presents its own uncertainty. A potential problem is that parents may lack knowledge about prenatal screening or newborn screening in general, and may be undergoing a stressful period, where it becomes difficult to fully comprehend the disclosed information and provide informed consent. While it may be a priority for prenatal screening, providing parents with information about newborn screening is not necessarily at the top of the list of what they need to know**.** Parents may not understand the need for screening for rare genetic disorders. Besides, even pediatricians and other healthcare providers who are caring for babies in the newborn period may have never seen many of these conditions, and do not fully understand the manifestations or health implications of these diseases. They may be unable to provide accurate information to parents when they have questions about prenatal genetic screening.

#### Uncertainty related to screening for late onset disorders prenatally or at birth

Additional ethical concerns relate to appropriateness of PGT or newborn screening for late-onset disorders. There may be opportunity to screen embryos during PGT (as in Huntington’s disease) or in the newborn period (such as for Pompe disease) [19] and other diseases in which a child can become symptomatic later on in life. Some affected individuals may have markers for diagnosis of the disease, but they may never become symptomatic. This raises ethical issues related to how much counseling for parents is adequate to enable them understand these complexities. The number of genetics professionals (both geneticists and genetic counselors) may not be adequate to cover the increased demand for services for newborn screening panels. This might delay diagnosis for more severe early-onset diseases, as focus is shifted to diagnosis of late-onset disorders (which may not be as severe or disabling, and may never manifest clinically).

### The moral significance of uncertainty

Decisions taken after PGT should put in consideration the implication of mosaicism at a given stage of development when biopsy is undertaken, the risk of the findings as to how they may affect clinical outcomes, financial implications and ability to counsel patients [31, 32]. Ultimately, the testing should be individualized to the needs of the couples. The moral significance of uncertainty is based on the concern whether moral judgment is accomplished by intuition or conscious reason [33, 34]. The extent to which conscious reasoning, as opposed to intuition, plays a role in determining moral judgment, and whether moral judgment is a controlled or an automatic process are issues relevant to uncertainty and ambiguity [33]. And this uncertainty is related to four major concerns about prenatal screening [34]. First, autonomy and respect for persons, the future autonomy of the child to determine whether to have the test is removed. Nonmaleficence, from potential harm to the child, and to the family, in screening for these late-onset disorders. An asymptomatic child that is screened and then confirmed positive for one of these disorders (or family) may suffer anxiety or stigma and discrimination.

There is tremendous potential benefit in prevention of hereditary genetic or chromosomal disorders [34]. The potential benefit for screening for late-onset disorders is to avoid diagnostic challenges which may occur when the affected individual develops unclear symptoms, leading to several diagnostic tests in order to help ascertain the cause of the patients’’ symptoms [34]. The counter argument is that it is ethically challenging to justify creating and destroying embryos for the purpose of testing for late-onset conditions, some of which may never manifest or occur much later in life [34]. Besides, parents’ options include whether or not to transfer all ‘unaffected’ embryos: noncarriers as well as carriers, yet carrier embryos are likely to develop into healthy individuals and selecting against them potentially stigmatizes carrier status [34]. If the carriers are at risk of developing some symptoms of the disorder, there is some justification to discard carrier status embryos [34]. For disorders whose effective treatment is not available, the benefit is unclear, and for screening geared primarily for sex selection for no medical reason, it is ethically debatable whether this may be justifiable [34]. Another area of concern is justice related to access to care and human resources needed to implement the testing.

#### Implications for the need for decision-support tools for patients and clients

Availability and use of prenatal diagnostic procedures have potential to improve the quality of prenatal care, prevent hereditary genetic and chromosomal disorders and improve parental reproductive choices and decision-making. This has several implications for decision-making support [33, 34]. First, there should be clear information packages to address uncertainty during counseling. The different techniques not only have false-positive rates, but also may be deleterious to embryo development, leading to miscarriage [33]. Secondly, should be clearly specified care pathways to aid decision-making, as well as practice guidelines and oversight to address the uncertainty parents and clinicians face when they use these technologies. Health professional education and public engagement efforts are critical for quality assurance in addressing the challenges and opportunities related to decision-making for using prenatal diagnostic procedures. Thirdly, the application of the new and higher performance technologies leads to identification of genetic variations, the biological and clinical importance of which may not sufficiently understood. Fourthly, there is also need to develop less invasive procedures to avoid embryo damage and wastage. Lastly, client concerns and values need to be incorporated in the decision-making process.

## Conclusion

PGT and other prenatal diagnostic procedures have potential for creating uncertainty as well as being used for ethically controversial conditions. Therefore, there is need for regulation and oversight, with clear protocols and guidelines of when and how different procedures could be performed as well as implications of the different decisions that patients may make. Professional self-regulation is preferable, and health professions’ societies must provide more definitive guidelines in order for regulation to be effective. Different health professionals, including infertility specialists, physicians, and embryologists, obstetrician-gynecologists, geneticists, and genetic counselors, need to meet, map the landscape for prenatal diagnostic testing and develop consensus-based guidelines on prenatal diagnostic procedures, based on the perceived needs of their clients, need to advance the professions and social values of their communities, in order to address the uncertainty related to prenatal diagnostic procedures.

## Data Availability

Not applicable.

## Abbreviations

AMH: Anti-Mullerian Hormone
CVS: Chorionic Villus Sampling
DNA: Deoxyribonucleic acid
HLA: Human Leukcocyte antigen
IVF: In vitro fertilization
PCR: Polymerase Chain reaction
PGD: Pre-implantation genetic diagnosis
PGD: Pre-implantation genetic testing
RHD: Rhesus Disease
RNA: Ribonucleic acid
PGS: Preimplantation genetic screening
PKU: Phenyl ketonuria
